# *FAT*10, a gene up-regulated in various cancers, is cell-cycle regulated

**DOI:** 10.1186/1747-1028-1-20

**Published:** 2006-09-08

**Authors:** Chuan-Bian Lim, Dongwei Zhang, Caroline GL Lee

**Affiliations:** 1 Division of Medical Sciences, National Cancer Center, Singapore; 2 Department of Biochemistry, Yong Loo Lin School of Medicine, National University of Singapore, Singapore

## Abstract

**Background:**

FAT10 is a member of the ubiquitin-like-modifier family of proteins. Over-expression of the FAT10 gene was observed in the tumors of several epithelial cancers. High FAT10 expression was found to lead to increased chromosome instability via the reduction in the kinetochore localization of MAD2 during the prometaphase stage of the cell-cycle. FAT10 expression was also previously reported to be regulated by cytokines and p53.

**Results:**

Here, we report that FAT10 expression is regulated at the protein and transcript level during cell-cycle with highest expression observed during the S-phase of the cell-cycle. The distal region between -1997 to -975 bp from the transcription start site of the FAT10 promoter may play a role in the repression of FAT10 expression during G2/M phase of the cell-cycle.

**Conclusion:**

FAT10 expression is regulated during cell-cycle.

## 

One of the hallmarks of cancer is the deregulation of cell-cycle controls [1,2]. The cell-cycle is the timed sequence of events by which a cell duplicates all its components and produces two daughter cells. Successful progression through the cell cycle requires sequential activation of a variety of catalytic subunits called cyclin-dependent kinases (Cdks) that are dependent on the periodic synthesis of cell-cycle specific regulatory subunits known as cyclins [3]. The orderly transitions between the cell cycle phases are regulated by the proteolysis of cyclins; inhibitory phosphorylation of both Cdks and cyclins; as well as by the interaction with inhibitory regulators in a timely fashion [3]. Proteolytic destruction of cyclin proteins occurs through the 26S proteasome pathway, a process whereby each protein is covalently conjugated to an ubiquitin chain at one or more lysine residues, which serves as the signal for targeting the protein to the 26S proteasome for degradation [3,4]. Defects in the ubiquitin system can lead to a plethora of diseases, including many cancers. For instance, MDM2, an ubiquitin ligase, was found to be amplified in human sarcomas, gliomas, breast carcinomas and leukemias [5-9], probably by deregulating the p53-dependent tumor suppression pathway [10].

While ubiquitin is a key regulator of the cell cycle, a growing group of related low-molecular-weight proteins known as ubiquitin-like proteins have been identified within the last few years that are implicated in diverse cellular processes. To date, two families of ubiquitin-like proteins are recognized, namely the ubiquitin-like modifiers (UBLs) and ubiquitin-domain proteins (UDPs) [11]. As their family name suggests, they have regions of homology with the 76 amino acids of the ubiquitin protein, but are otherwise unrelated to each other. Like ubiquitin, the UBL family, for example, SUMO/Sentrin and NEDD8/Rub1, function as modifiers by conjugation to other proteins. The UDP proteins, such as RAD23 and Elongin B, on the other hand, are not covalently attached to target proteins although they contain ubiquitin homology domains [11].

FAT10, also known as diubiquitin, belongs to the UBL class of ubiquitin-like proteins. Initially identified as one of the genes at the major histocompatibility complex locus in human chromosome 6 [12], the *FAT*10 gene encodes an 18 kDa protein containing 165 amino acid residues that shares 29% and 36% identity with ubiquitin at the N- and C-terminus, respectively. Although its function has not been fully elucidated, it has been implicated to play important roles in various cellular processes.

Like ubiquitin [13], FAT10 also functions as a proteinaceous tag, which when attached to a substrate protein, targets it to the 26S proteasome for degradation [14]. While ubiquitin is recycled from the degraded target proteins, FAT10 has a relatively short half-life and is degraded together with its target [14]. The binding of the NEDD8 ultimate buster 1 long (NUB1L) protein to FAT10 was found to further accelerate the degradation of FAT10 [15].

Knockout of the FAT10 gene in mice causes minimal phenotypic changes. These mice exhibit increased sensitivity to endotoxin challenge and their lymphocytes are more susceptible to spontaneous apoptotic death [16].

On the other hand, over-expression of the FAT10 gene was observed in the tumors of several cancers including gastrointestinal and gynecological cancers [17]. High level of the FAT10 protein in cells was recently found to lead to increased mitotic non-dysjunction and chromosome instability [18]. FAT10 was shown to interact with the mitotic checkpoint protein, MAD2, during the mitotic phase of the cell-cycle [18]. An abbreviated mitotic phase and a reduction in the kinetochore localization of the MAD2 protein during the prometaphase stage of the cell-cycle was also observed in cells expressing high levels of the FAT10 protein [18].

Expression of the FAT10 gene was reported to be up-regulated by cytokines IFN-γ and TNF-α [12,19,20]. FAT10 expression was recently shown to be negatively regulated by p53 [21], which plays important roles in the regulation of the cell-cycle [22]. The abnormally high expression of FAT10 in the tumors of several cancers, coupled with the observation that high FAT10 levels in cells lead to mitotic non-dysjunction and chromosome instability through the reduction of the MAD2 kinetochore localization during the pro-metaphase stage of the cell-cycle; as well as the findings that its expression is positively regulated by TNF-α, a presumptive tumor promoter [14,23], but negatively regulated by p53, the "guardian-of-the-genome" [24], together, strongly implicate the important role that FAT10 plays in cell-cycle control and tumorigenesis. Since many genes that are involved in cell-cycle regulation are, themselves, regulated in a cell-cycle dependent fashion [25,26], in this study, we examine if the expression of the FAT10 gene is also regulated in a cell-cycle dependent manner.

## Results and discussion

### FAT10 expression in HCT116 human colon carcinoma cells is cell cycle-regulated at the transcript level

**Figure 1:**
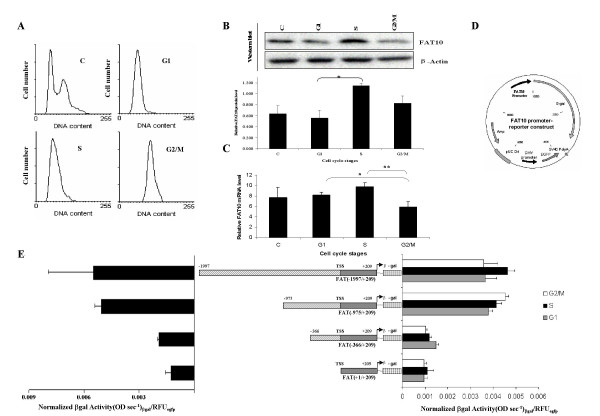
**FAT10 expression at the transcript level and promoter is cell-cycle regulation in HCT116 cells**. (A) Cell-cycle profiles of HCT116 cells synchronized at the various cell-cycle stages: C – Asynchronous cells; G1-Cells synchronized to G1-phase with L-minosine; S-Cells syncrhonized to S-phase with Thymidine and G2/M-Cells synchronized to G2/M phase with nocodazole. (B) FAT10 protein expression normalized against β-actin protein levels. Top panel: Western blot of a representative experiment. Bottom panel: mean and standard deviation of results quantitated from Western blots from three independent experiments. *p < 0.05. (C) FAT10 mRNA transcript levels normalized against β-actin mRNA levels. Results shown are the mean and standard deviation from three independent experiments. *p < 0.05 and **p < 0.01. (D) Promoter-reporter construct used to assay FAT10 promoter activity. Various lengths of the FAT10 promoter drive the β-galactosidase reporter gene while the constitutive CMV promoter drives the EGFP to normalize for differences in transfection efficiencies. (E) Left panel – FAT10 promoter activity in HCT116 cells expressed as normalized β-galactosidase activity from 3 independent experiments. Middle Panel – Pictorial representation of the various regions of DNA upstream and downstream the transcription start site of the FAT10 gene that is cloned before the β-galactosidase gene. Right Panel – FAT10 promoter activity in HCT116 cells synchronized to the various cell-cycle stages (white: G2/M; black: S and Grey: G1) in HCT116 cells. Results shown are the mean and standard deviation from three independent experiments.

The finding that the FAT10 gene expression is negatively regulated by p53 [21], which is known to play an important role in cell-cycle regulation; coupled with the observation of the over-expression of the FAT10 gene in the tumors of various cancers [17], as well as the demonstration that abnormally high levels of FAT10 in HCT116 cells can disrupt cell-cycle controls leading to chromosome instability [18], suggested that the endogenous *FAT*10 expression may be regulated throughout the normal cell cycle. To address if the FAT10 gene is itself regulated in a cell-cycle dependent manner, populations of HCT116 cells were blocked at G1, S, or M phase of the cell cycle and the expression of the FAT10 gene at the protein and transcript levels were examined by Western blot analyses and reverse-transcription (RT) real-time PCR. The cell cycle stage of each HCT116 population was determined by flow cytometric analysis of the DNA content in the cell population (Fig. 1A). As shown in Fig. 1B, FAT10 protein expression peaks in S phase of the cell cycle and then decreased when cells were arrested at the G2/M border in nocodazole. Similar and more statistically significant trends were observed at the transcript/mRNA level (Fig. 1C). The observation of lower FAT10 expression at the G2/M border of the cell-cycle stage is consistent with parental HCT116 cells maintaining low levels of FAT10 during the G2/M phase of the cell-cycle to preserve chromosome stability by preventing FAT10 from interacting with MAD2 and reducing the kinetochore localization of MAD2 during this stage of cell-cycle which may lead to mitotic non-dysjunction and chromosome instability.

### The promoter of FAT10 plays a role in the cell-cycle-regulated expression of FAT10

Since FAT10 expression was found to be cell-cycle regulated at the transcript level, we examined the promoter of FAT10 to determine if it plays a role in the cell-cycle regulation of FAT10 expression and to delineate the region of the promoter that may be responsible for the cell-cycle regulation of FAT10 expression. We previously delineated the regions of the FAT10 promoter that mediate promoter activities in Hep3B, HepG2 and KB3-1 cells [21]. In this study, we examined FAT10 promoter activities in HCT116 cells whose FAT10 expression is cell-cycle regulated at both the protein (Fig. 1B) and the transcript levels (Fig. 1C) and which we previously reported that artificial over-expression of the FAT10 gene in these cells results in chromosome instability [18]. As evident in Figure 1E (left panel), maximum FAT10 promoter activity was observed in HCT116 cells when FAT(-1997/+209) and FAT(-975/+209) promoter region was cloned upstream the β-galactosidase reporter and significantly lower promoter activity was found when FAT(-366/+209) and FAT(+1/+209) was cloned upstream the reporter. Hence, the trend of FAT10 promoter activities observed for HCT116 cells were similar to those previously reported for Hep3B, HepG2 and KB3-1 cells although the absolute promoter activity in HCT116 which is p53^+ ^was more similar to p53^+^HepG2 and KB3-1 cells and approximately 22–30 fold lower than the FAT10 promoter activity in p53^- ^Hep3B cells [21].

To determine the region of the FAT10 promoter that may mediate cell-cycle regulation, the four constructs carrying different lengths of the FAT10 promoter upstream the β-galactosidase reporter were transfected into HCT116 cells and treated with mimosine, thymidine, or nocodazole to arrest them in G1, S or M phase, respectively, 24 h later. Flow cytometric analyses of DNA were used to confirm the cell-cycle stage of the transfected cells (Fig 1A). Similar to our observation of FAT10 mRNA expression at the different phase of the cell-cycle, when the longest promoter fragment (FAT(-1997/+209)) was cloned upstream the β-galactosidase reporter, highest FAT10 promoter activity was observed in S-phase but not in G1 or G2/M phases (Fig. 1E, right panel). However, when the distal promoter region was deleted to -975 bp from the transcription start site (TSS) (FAT(-975/+209)), the repression of the FAT10 promoter activity at the G2/M phase of the cell-cycle was relieved while its activity at the G1 phase remain low (Fig. 1E, right panel). Hence, the distal promoter region between -1997 and -975 bp from the TSS may play a role in the inhibiting FAT10 expression during the G2/M phase of the cell-cycle.

In light of our earlier observations that over-expression of the FAT10 gene in HCT116 cells will result in the reduction of the kinetochore localization of the important checkpoint MAD2 protein leading to mitotic non-dysjunction and chromosome instability [18], our current observation that FAT10 expression, through the repression of the FAT10 promoter activity, is kept low during G2/M phase of cell-cycle is notable. It suggests that cells may have evolved to ensure the fidelity of its cell-division by repressing the expression of proteins like FAT10 which might interact and interfere with the functions of important checkpoint proteins like MAD2 during the G2/M phase of the cell-cycle. Repression of the FAT10 gene is observed at two levels, the first general major repression is carried out by the p53 protein [21] and the second more specific repression is observed at the G2/M phase of the cell-cycle where FAT10 was shown to interact with mitotic checkpoint protein, MAD2. De-regulation of the expression of these proteins like FAT10 would result in the de-regulation of the G2/M cell-cycle phase controls resulting in the abberant distribution of chromosomes during cell-division, hence, leading to tumorigenesis.

## Materials and methods

### Cell lines and tissue culture

HCT116 (colon carcinoma) cells were purchased from American Type Culture Collection and grown in McCoy's 5A (Sigma) supplemented with 10% fetal calf serum at 37°C in a humidified atmosphere of 5% CO_2_.

### Cell cycle synchronization and flow cytometry

Synchronization of HCT116 cells at the various cell cycle stages was performed according to a protocol described by Su *et al *[27]. Briefly, cells were initially synchronized to the same cell-cycle phase by incubating them with 3 mM thymidine (Sigma-Aldrich) in McCoy's 5A medium. After 17 h incubation at 37°C, the cells were washed twice with PBS and incubated in normal medium for an additional 12 h at 37°C to release them from the block. The cells were then enriched for G1, S and GM phases by carrying out the following steps: G1 (0.5 mM L-minosine (Sigma-Aldrich) for 15 h); S phase (3 mM thymidine for 16 h); and M phase (100 ng/ml nocodazole (Sigma-Aldrich) for 12 h). Cells were treated with trypsin or 4 mM EDTA and fixed in 1% paraformaldehyde overnight at 4°C for flow cytometry. DNA was stained with 0.01 mg/ml propidium iodide in PBS containing 0.1% Triton X-100 and 0.1 mg/mL RNase A for 1 h at 37°C in the dark. Stained cells were analyzed using the FACScalibur™ flow cytometer (BD Biosciences, Franklin Lakes, NJ).

### Western blot

Western blot analyses was performed on HCT116 cell lysates (7 μg/well) (Lee et al., 2003) using the rabbit anti-FAT10 (Lee et al., 2003), and goat anti-actin (Santa Cruz Biotechnology) primary antibodies and HRP-conjugated anti-mouse or anti-goat IgG secondary antibodies (Pierce Biotechnology). The Western blot membranes were visualized by ECL Advance^™ ^Western blotting detection kit (Amersham Biosciences). Results were quantified through densitometric analysis.

### Quantitative real-time RT-PCR analysis

Total cellular RNA was isolated from HCT116 cells using an RNeasy mini-kit (Qiagen). Quantitation of *FAT*10 and β-actin transcript levels was performed as previously reported [21]. Briefly, cDNA was synthesized from total RNA using Superscript II reverse transcriptase (Invitrogen) and real-time polymerase chain reaction (PCR) was performed on the cDNA products in a Rotor-Gene 2000 Real-Time Thermal Cycler (Corbett Research) using the QuantiTect™ SYBR^® ^Green PCR Kit (Qiagen) and the following primers: *FAT*10 (F: 5'CAATGCTTCCTGCCTCTGTG, R: 5'TGCCTCTTTGCCTCATCACC); β-actin (F: 5'ATGTTTGAGACCTTCACACC, R: AGGTAGTCAGTCAGGTCCCGGCC). The PCR reaction for FAT10 comprise of a denaturation step at 95°C for 15 min followed by 45 cycles of amplification at 95°C for 30 sec, 64°C for 30 sec and 72°C for 30 sec, while β-actin amplification involved an initial denaturation at 95°C for 15 min followed by 40 cycles at 95°C for 30 sec, 55°C for 30 sec and 72°C for 30 sec. SYBR^® ^Green fluorescence was measured after each extension step. Standard curves for *FAT*10 and β-actin were generated using serial dilution of plasmids containing the respective cDNAs. The linear range for all the respective gene expression was determined to be between 10^3 ^to 10^8 ^copies (r^2 ^for FAT10 is 0.9993; and β-actin is 0.992 respectively). *FAT*10 expression was normalized against the housekeeping β-actin gene expression. All experiments were performed in triplicate.

### Construction of reporter plasmids

A series of different length fragments upstream the TLSS/TSS of the *FAT*10 gene (+1/+209, -366/+209, -975/+209 and -1997/+209) were cloned into an expression vector (pFAT10-EGFP) whereby the *FAT*10 promoter drives the β-galactosidase (β-gal) reporter gene while the constitutive cytomegalovirus (CMV) promoter drives the enhanced green fluorescent protein (EGFP) reporter gene (Fig. 1D) as described previously [21]. All constructs were sequenced to exclude any PCR errors. Plasmid DNA was prepared using an endotoxin-free Maxi Prep kit (Qiagen).

### Transient transfection and β-galactosidase reporter assays

Two hundred thirty thousand HCT116 cells were seeded onto each well of a 6-well plate the day before transfection. Transfection was carried out using Superfect reagent (Qiagen) according to the manufacturer's instructions. After 24-h incubation, cells were enriched for G1, S and M phases by carrying out the steps as described above. Cells were then washed with PBS and solubilized in 1x reporter lysis buffer (Promega) and β-galactosidase activity was determined in a kinetic assay as described previously [21].

### Statistical and computational methods

Statistically significant differences between samples means were determined by single-factor analysis of variance (ANOVA) followed by a post-hoc Tukey's test for multiple pairwise comparisons. *P *< 0.05 was considered significant.

## Competing interests

To the best of our knowledge, there is no possible conflict of interests for any of the above authors.

There are also financial disclosures to be made by any of the authors as this work is funded from a grant to CG Lee from the Academic Research Fund, National University of Singapore as well as block funding to CG Lee laboratory at the National Cancer Centre, Singapore.

## Authors' contributions

CBL carried out the cell-culture and promoter-reporter experiments and wrote the first draft of the manuscript. DWZ engineered the promoter-reporter constructs and mentored CBL on the cell-culture and promoter-reporter assays. CGL conceived of the study, and participated in its design and coordination. CGL wrote the final version of the manuscript. All authors read and approved the final manuscript.
